# Sex-Dependent Performance of the Neutrophil-to-Lymphocyte, Monocyte-to-Lymphocyte, Platelet-to-Lymphocyte and Mean Platelet Volume-to-Platelet Ratios in Discriminating COVID-19 Severity

**DOI:** 10.3389/fcvm.2022.822556

**Published:** 2022-04-08

**Authors:** Martha Fors, Santiago Ballaz, Hegira Ramírez, Francisco X. Mora, Mary Pulgar-Sánchez, Kevin Chamorro, Esteban Fernández-Moreira

**Affiliations:** ^1^Escuela de Medicina, Universidad de las Américas-UDLA, Quito, Ecuador; ^2^School of Biological Sciences and Engineering, Universidad Yachay Tech, Ibarra, Ecuador; ^3^Universidad Espíritu Santo, Samborondón, Ecuador; ^4^Universidad Ecotec, Samborondón, Ecuador; ^5^IESS Hospital Quito Sur, Quito, Ecuador; ^6^School of Biological Sciences and Engineering, Universidad Yachay Tech, Urcuquí, Ecuador; ^7^School of Mathematics and Computational Sciences, Universidad Yachay Tech, Urcuquí, Ecuador; ^8^Facultad de Medicina, Universidad de Especialidades Espíritu Santo, Samborondón, Ecuador

**Keywords:** COVID-19, neutrophil-to-lymphocyte ratio, platelet-to-lymphocyte ratio, monocyte-to-lymphocyte ratio, mean platelet volume-to-platelet ratio, gender

## Abstract

**Background:**

The neutrophil-to-lymphocyte ratio (NLR), platelet-to-lymphocyte ratio (PLR), lymphocyte-to-monocyte ratio (LMR), and mean platelet volume-to-platelet ratio (MPR) are combined hematology tests that predict COVID-19 severity, although with different cut-off values. Because sex significantly impacts immune responses and the course of COVID-19, the ratios could be biased by sex.

**Purpose:**

This study aims to evaluate sex-dependent differences in the contribution of NLR, PLR, MLR, and MPR to COVID-19 severity and mortality upon hospital admission using a sample of pneumonia patients with SARS-CoV-2 infection.

**Methods:**

This single-center observational cross-sectional study included 3,280 confirmed COVID-19 cases (CDC 2019-Novel Coronavirus real-time RT-PCR Diagnostic) from Quito (Ecuador). The receiver operating characteristic (ROC) curve analysis was conducted to identify optimal cut-offs of the above parameters when discriminating severe COVID-19 pneumonia and mortality risks after segregation by sex. Severe COVID-19 pneumonia was defined as having PaO_2_ < 60 mmHg and SpO_2_ < 94%, whereas non-severe COVID-19 pneumonia was defined as having PaO_2_ ≥ 60 mmHg and SpO_2_ ≥ 94%.

**Results:**

The mortality rate of COVID-19 among men was double that in women. Severe COVID-19 pneumonia and non-surviving patients had a higher level of NLR, MLR, PLR, and MPR. The medians of NLR, MLR, and MPR in men were significantly higher, but PLR was not different between men and women. In men, these ratios had lower cut-offs than in women (NLR: 2.42 vs. 3.31, MLR: 0.24 vs. 0.35, and PLR: 83.9 vs. 151.9). The sensitivity of NLR, MLR, and PLR to predict pneumonia severity was better in men (69–77%), whereas their specificity was enhanced in women compared to men (70–76% vs. 23–48%).

**Conclusion:**

These ratios may represent widely available biomarkers in COVID-19 since they were significant predictors for disease severity and mortality although with different performances in men and women.

## Introduction

Combined hematology tests, such as the neutrophil-to-lymphocyte ratio (NLR), platelet-to-lymphocyte ratio (PLR), and monocyte-to-lymphocyte ratio (MLR), have become promising indicators of disease severity in patients with COVID-19 (1–7). In addition, the mean platelet volume (MPV), a hallmark of platelet activation and largely a high MPV/platelet count ratio (MPR), which predict long-term mortality in patients suffering from some cancers (8, 9), also constitute risk factors for severe pneumonia in patients suffering from COVID-19 (10). Their performance in predicting severity and mortality in patients with COVID-19 should then be secured in order to make medical care decisions (4). A limitation of these ratios is that they not only show ethnic differences (11, 12) but also they can be deeply influenced by sex (13), whose dependence has not yet been explored in COVID-19 disease.

Worldwide statistics currently show that COVID-19 severity is sex-dependent since more men than women die from SARS-CoV-2 infection (14–16). Animal studies have confirmed the sex-dependent susceptibility to SARS-CoV-2 and severity of lung illness (17). Estrogen and progesterone seem to provide protection to women against COVID-19 (18, 19). In contrast, sex-biased expression of ACE2, coupled with the regulation of TMPRSS2 by androgens, increases SARS-CoV-2 susceptibility in men compared to women (20). A valid hypothesis is that women express more toll-like receptor 7 (TLR7), which is encoded on the X chromosome. Because TLR7 detects viral single-strand RNAs, the innate immune response of women to SARS-CoV-2 seems to be more robust in women (21). Moreover, sex differences in platelet TLRs are likely to contribute to differences in COVID-19 severity (22). The revealing of hematological parameters meaningful to the innate immune response segregated by sex will undoubtedly help develop better treatment and prevention strategies against COVID-19.

Ecuador is among the top ten most hit countries by COVID-19 in Latin America (766,398 cases and 34,730 deaths as of February 7, 2022) because the public health sector was one of the losers of the austerity program cutting off resources imposed by the 2019 economic crisis. In developing countries like Ecuador, where hospital resources and personnel are limited, the evaluation of the status of patients with COVID-19 on admission is critical for their clinical management. In this vein, the refinement of combined hematology and laboratory tests as reliable biomarkers of COVID-19 severity is a must. The aim of this study was to evaluate sex differences in the contribution of the NLR, PLR, MLR, and MPR parameters to severe COVID-19 pneumonia and mortality using a sample of patients with COVID-19 infected with SARS-CoV-2. The role of applicable cut-offs of these ratios was investigated using the receiver operating characteristic (ROC) curve analysis for evaluating the discriminatory power of COVID-19 severity of these combined hematology parameters.

## Materials and Methods

### Design

This observational retrospective study included 3,280 patients with COVID-19 admitted at the IESS Sur Hospital in Quito, Ecuador, at the beginning of the coronavirus pandemic (from March 13 to June 17, 2020). The subjects included in the study were confirmed cases of COVID-19 disease over 18 years old who had a pneumonia severity index (PSI) > 3 and arterial blood gasometry exam during the triage evaluation (23). Individuals with a PSI ≤ 3 did not undertake a gasometry exam and were excluded. A confirmed COVID-19 case was defined as a subject suffering from COVID-19-like symptoms and at the same time having a positive result in the real-time reverse transcriptase-polymerase chain reaction (RT-PCR) test for SARS-CoV-2 (CDC 2019-Novel Coronavirus Real-Time RT-PCR Diagnostic Panel in upper and lower respiratory specimens). Patient categorization was conducted at the time of hospital admission in agreement with the NIH guidelines for the severity of COVID-19 pneumonia.^1^ Patients with COVID-19 were categorized as severe (PaO_2_ < 60 mmHg; SpO_2_ < 94%) and non-severe (PaO_2_ ≥ 60 mmHg; SpO_2_ ≥ 94%). This classification allowed us to predict the adverse clinical outcomes of COVID-19-induced pneumonia.

### Hematology Tests

Hematology analyses were performed using a Sysmex XN-550™ Hematology Analyzer (Sysmex America Inc., United States). Arterial blood gasometry was conducted on a RAPIDPoint® 500 blood gas system (Siemens Healthcare GmbH; Germany) under a controlled atmosphere. The calculation of the ratios was as follows: NLR, absolute neutrophil count divided by the absolute lymphocyte count; MLR, absolute monocyte count divided by absolute lymphocyte count; PLR, platelet count divided by absolute lymphocyte count; and MPR, mean platelet volume divided by platelet count.

### Statistical Analysis

Statistical analysis was performed using SPSS v24.0 for Windows (SPSS Inc., Chicago, IL, United States). Data were expressed as either mean and standard deviation or median and interquartile range (IQR) for continuous variables or as absolute counts and percentages for categorical variables. A chi-square test was run to challenge the association between severity, deaths, and sex. The Wilcoxon rank-sum test was used to compare medians of ratios between the non-severe and severe groups, between sex (women and men), and related to death (yes or no). Comparisons across independent experimental groups were performed using the Kruskal–Wallis *H* test followed by Dunn’s *post hoc* test for continuous data. A two-sided *p*-value < 0.05 was considered statistically significant. The performance of the ratios in discriminating severe from non-severe cases was assessed by areas under the curve (AUC) of ROC curves, which showed the relationship between sensitivity and 1-specificity. The point of the curves with both the maximum sensitivity and specificity was selected as the optimal cut-off point using the Youden Index. Parameters with an area under the curve (AUC) < 0.55 were not acceptable.

### Ethics

The STROBE guidelines were followed to conduct this study. The evaluation of a Human Research Ethics Committee was not required given the retrospective nature of the study, which consisted of non-experimental research with secondary data, anonymized patients, type and design with descriptive characteristics. The Infectious Disease Prevention and Epidemiological Surveillance Unit of the IESS Hospital Quito Sur gave the downloading authorization from the digital clinical records system in accordance with the policies established by the Ecuadorian government.

## Results

A sample of 3,280 confirmed patients with COVID-19 was included in this study. The median age for women was 41 (IQR: 31), whereas for men it was 39 (IQR: 30). No significant statistical differences were found for age between men and women (Wilcoxon rank-sum test *p* = 0.28). Neither were there significant differences in the numbers of men and women when distributed by groups of age in the sample (Wilcoxon rank-sum test *p* = 0.53). Sex-based distribution of patients across the levels of severity, mortality, and age groups is shown in Table 1. There were 635 (19.4%) severe cases and 2,645 (80.6%) non-severe cases of COVID-19 pneumonia. Mortality reached 3.1% of the sample. Approximately two-thirds of the deaths were men (69.9%). There were statistically significant differences in both severities of pneumonia and in the percentage of deceases as a function of the sex.

**TABLE 1:** Sex-dependent distribution of severity, mortality and age in COVID-19 patients.

	Women *n* = 1637 (49.9%)	Men *n* = 1643 (50.1%)	Total *n* = 3280 (100.0%)	
	*n* (%)	*n* (%)	*n* (%)	*p*-value*
**Severity**				
Severe	290 (17.1)	345 (21.0)	635 (11.9)	<0.001
Non-Severe	1493 (91.2)	1398 (85.1)	2891 (88.1)	
**Death**				
Yes	34 (2.1)	69 (4.2)	103 (3.1)	<0.001
No	1603 (97.9)	1574 (95.8)	3177 (96.9)	
**Age**				
18–40	817 (49.9)	852 (51.9)	1669 (50.9)	
41–64	527 (32.2)	509 (31.0)	1036 (31.6)	0.53
≥65	293 (17.9)	282 (17.2)	575 (17.5)	

*Chi-square test.

Table 2 shows the median and IQR of NLR, MLR, PLR, and MPR in patients with COVID-19 grouped by the severity of COVID-19 pneumonia, sex, and mortality. NLR, MLR, and PLR levels were significantly higher in the severe group, whereas MPR levels were significantly higher in the non-severe group. NLR, MLR, and PLR levels were higher in men compared to women. In individuals who died from COVID-19, NLR and MLR levels were significantly higher compared to those who survived. Table 3 shows the median and IQR of NLR, PLR, and MPR in patients with COVID-19 grouped by age. It is observed that there are no differences in the MPR ratio among groups (K–W = 1.82, *p* = 0.78). Dunn’s *post hoc* analysis showed differences in NLR, MLR, and PLR levels between 18–40 and 41–64 years.

**TABLE 2:** Distribution of combined hematology tests among patients with COVID-19 based on severity, sex, and mortality.

Ratio	Median (IQR)	Median (IQR)	*p*-value*
**Severity**	Severe *n* = 635	Non-Severe *n* = 2645	
Neutrophil-to-lymphocyte ratio (NLR)	2.95 (3.65)	2.38 (2.80)	<0.001
Monocyte-to-lymphocyte ratio (MLR)	0.41 (0.31)	0.27 (0.20)	<0.001
Platelet-to-lymphocyte ratio (PLR)	106.9 (102.8)	97.4 (80.6)	0.001
Mean platelet volume/platelet count ratio (MPR)	0.034 (0.018)	0.031 (0.015)	<0.001
**Sex**	Women *n* = 1637	Men *n* = 1643	
Neutrophil-to-lymphocyte ratio (NLR)	2.35 (2.48)	2.693 (3.57)	<0.001
Monocyte-to-lymphocyte ratio (MLR)	0.25 (0.16)	0.301 (0.25)	<0.001
Platelet-to-lymphocyte ratio (PLR)	98.99 (75.84)	100.01 (94.02)	0.780
Mean platelet volume/platelet count ratio (MPR)	0.030 (0.014)	0.033 (0.014)	<0.001
**Death**	Yes *n* = 103	No *n* = 3177	
Neutrophil-to-lymphocyte ratio (NLR)	9.00 (10.33)	2.44 (2.79)	<0.001
Monocyte-to-lymphocyte ratio (MLR)	0.55 (0.66)	0.27 (0.20)	<0.001
Platelet-to-lymphocyte ratio (PLR)	211.2 (27.3)	98.02 (80.4)	<0.001
Mean platelet volume/platelet count ratio (MPR)	0.040 (0.02)	0.031 (0.01)	<0.001

*Wilcoxon rank-sum test.

**TABLE 3:** Distribution of combined hematology tests across age groups of patients with COVID-19.

Ratio	Median (IQR)	Median (IQR)	Median (IQR)	*p*-value*
	18–40 years	41–64 years	≥65 years	
NLR	2.42 (2.71)	2.38 (2.81)	3.12 (3.9)	<0.001
MLR	0.27 (0.20)	0.27 (0.20)	0.30 (0.25)	0.001
PLR	96.8 (76.6)	91.41 (83.68)	120.0 (104.8)	0.001
MPR	0.031 (0.015)	0.032 (0.017)	0.031 (0.014)	0.784

*Kruskal–Wallis analysis.

**FIGURE 1:**
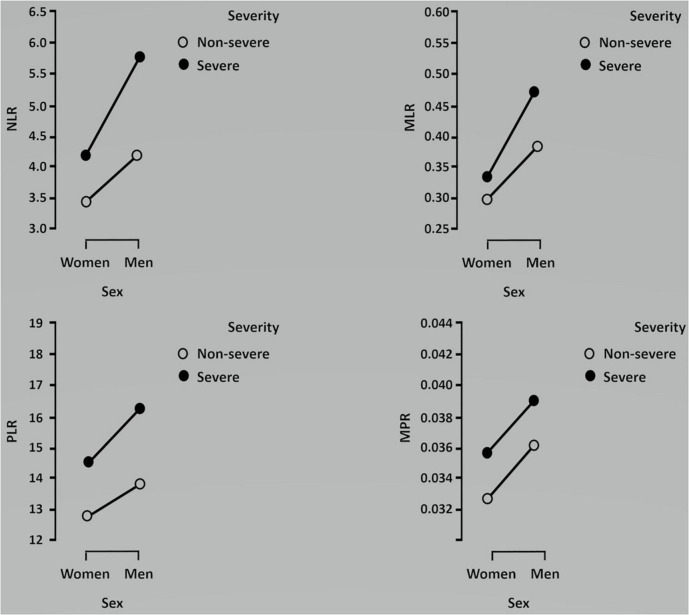
Kruskal–Wallis analysis comparing the association between each ratio and severity in men and women. Data are shown as medians.

**FIGURE 2:**
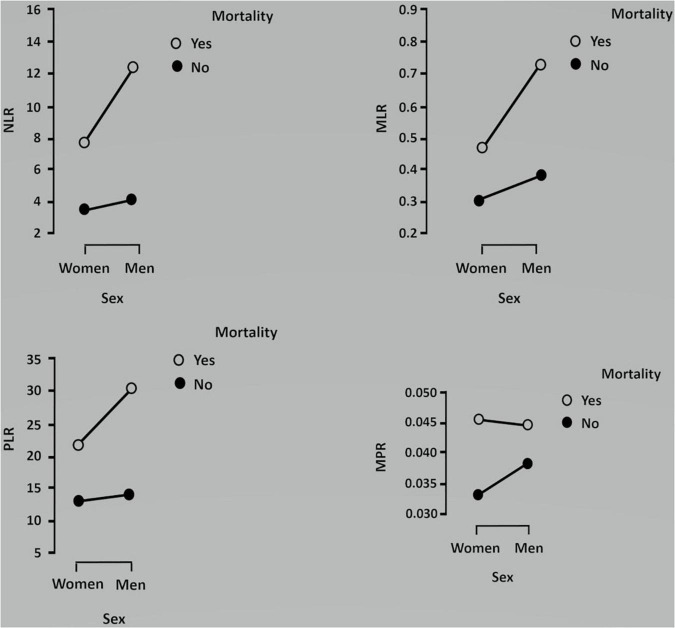
Kruskal–Wallis analysis comparing the association between each ratio and mortality in men and women. Data are shown as medians.

In Figure 1, NLR, MLR, PLR, and MPR levels were compared across COVID-19 pneumonia severity and sex using the Kruskal–Wallis analysis. Simple main effects analysis showed that severity was associated with higher NLR, MLR, PLR, and MPR levels, largely in men (*p* < 0.001). Dunn’s *post hoc* test revealed that all the comparisons were significant except for NLR and MPR levels in women in the severe group compared to men in the non-severe group, and for MLR level in women in the non-severe group vs. women in the severe group. Regarding PLR levels, no differences were found in any comparisons. The dependent NLR, MLR, PLR, and MPR variables were also compared across mortality and sex using the Kruskal–Wallis analysis, which gave statistically significant differences (Figure 2). Dunn’s *post hoc* tests revealed that most of the groups differed statistically (*p* < 0.001). There were no differences in NLR levels between surviving women and men, in MLR levels between non-surviving women and surviving men, in PLR levels between men and women, and in MPR levels between non-surviving women and non-surviving men.

**FIGURE 3:**
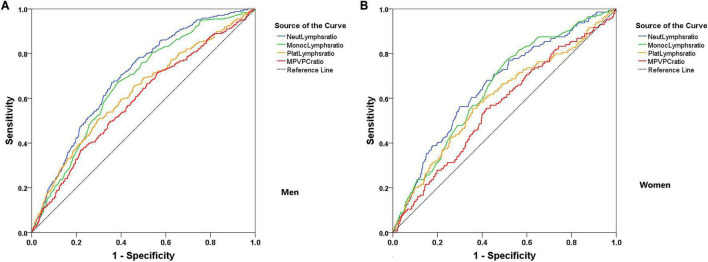
Receiver-operating characteristic (ROC) curves for the combined hematology tests evaluation of COVID-19 pneumonia severity segregated by sex. **(A)** Men and **(B)** Women.

Finally, data were submitted to AUC–ROC analysis (Figure 3) to determine the sensitivity and specificity of the hematology ratios in predicting severe COVID-19 pneumonia and their cut-offs. Most of the AUCs were above the acceptable threshold (0.55). The AUCs for the combined hematology tests were modest, ranging from 0.50 to 0.59 in the whole sample (Table 3). Except for MPR, the cut-offs of all the ratios in women were higher compared to men. NLR had a cut-off point of 2.42 in men vs. 3.34 in women (38% higher), while the cut-off points of MLR and PLR in women were 46 and 81% higher than in men, respectively. The sensitivity of NLR, MLR, and PLR was higher in men, whereas their specificity was superior in women. For instance, the sensitivity of PLR in men was 71 vs. 37% in women. The specificity of MLR in women was 61 vs. 23% in men. Unexpectedly, no sex differences were found in the cut-off point, sensitivity, and specificity of MPR (Table 4).

**TABLE 4:** Area under the curve-receiver operating characteristic (AUC-ROC) curve analysis for the combined hematology tests evaluation of COVID-19 pneumonia severity.

	AUC (95% CI)	Cut-off	Sensitivity	Specificity	*p*-value
**General**					
NLR	0.57 (0.55–0.60)	2.28	0.66	0.48	<0.001
MLR	0.57 (0.54–0.59)	0.33	0.48	0.63	<0.001
PLR	0.54 (0.51–0.56)	196.7	0.58	0.49	<0.001
MPR	0.55 (0.53–0.58)	0.03	0.43	0.64	<0.001
**Female**					
NLR	0.55 (0.51–0.58)	3.34	0.56	0.70	<0.001
MLR	0.53 (0.49–0.57)	0.35	0.52	0.61	<0.001
PLR	0.52 (0.48–0.55)	151.9	0.37	0.76	0.27
MPR	0.54 (0.50–0.57)	0.03	0.54	0.56	0.03
**Male**					
NLR	0.59 (0.56–0.63)	2.42	0.69	0.48	<0.001
MLR	0.59 (0.56–0.62)	0.24	0.77	0.23	0.05
PLR	0.56 (0.52–0.59)	83.9	0.71	0.41	<0.001
MPR	0.56 (0.52–0.59)	0.03	0.60	0.49	<0.001

## Discussion

This observational retrospective investigation was the first to report the cut-off values of the NLR, LMR, PLR, and MPR indices in patients with COVID-19 segregated by sex since most of the patients who died from COVID-19 were men. The performance of these biomarkers, which provide insight into COVID-19 progression and predictions of its severity, presented significant sex-based differences regardless of age. Our data pointed to the influence of sex in the inflammatory response to COVD-19.

The mortality rate in our sample was within the range of other reports (24, 25). Severe subjects had a higher NLR compared to non-severe patients, similar to the results obtained by others (26–28), and above the average (3.27, 95% CI: 1.99–4.55) reported in a meta-analysis (29). However, the sensitivity (66%) and specificity (48%) were fair for this indicator and were reported below the levels for these parameters elsewhere (30–32). Despite this, neutrophilia represents the hallmark of severe COVID-19, whereas lymph cell percentage is inversely related to its progression (32, 33). Because high NLR levels are positively correlated with mortality by COVID-19 (4–6, 34), a higher NLR value in our sample was still indicated to differentiate those subjects at risk of dying from COVID-19, especially if they are men (69% sensitivity).

The MLR biomarker was selected because of its prognostic value for Middle East Respiratory Syndrome Coronavirus (ıMERS-CoV) infection (35). A cut-off of 0.33 for MLR could discriminate patients with severe COVID-19 from those patients with non-severe COVID-19 with approximately 50% of sensitivity and specificity values in agreement with previous studies, yet with a higher cut-off (1). Compared to the work of Peng et al. (36), our AUC for MLR was lower. Surprisingly, the sensitivity of MLR to discriminate against severe COVID-19 subjects rose to 77% in men, yet with a specificity of 23% (61% in women). The PLR parameter reveals changes in platelet and lymphocyte counts because of acute inflammatory and pro-thrombotic conditions (37). PLR levels associated with severe COVID-19 were within the range since they were either higher (1, 26, 38) or lower (30) than the reference. PLR was higher in men, with a sensitivity of 71% (only 37% in women) and a specificity of 41% (76% in women), which was suggestive of a different cytokine storm in patients with COVID-19 (39) pending on the sex.

The MPR parameter has recently received attention as a prognostic marker in COVID-19 pneumonia (10). MPR reflects the proliferation of megakaryocytes and platelet production in the bone marrow (40). Patients with COVID-19 often have mild thrombocytopenia and appear to have increased platelet consumption, together with a corresponding increase in platelet production (41). Although men showed a lower MPR level compared to women and it would then be thought a relationship with a higher risk of dying from COVID-19, the AUC–ROC did not detect any sex differences in predicting severity. Accordingly, the relationship between MPR and COVID-19 severity remains ambiguous. More research is needed to define the MPR cut-off point, sensitivity, and specificity.

The AUC–ROC analysis revealed a fair performance of the combined hematology biomarkers in predicting COVID-19. Although it improved when the sample was split by sex, one should be careful not to generalize the AUC value as the only measure of test utility (42). There is no doubt that patients with serious COVID-19 have a dysregulated resistance reaction that permits viremia, thus ensuing hyper-inflammation and cytokine storm. Neutrophilia is the expression of the cytokine reaction and a hyper-immunity in this disease (43). Sex-driven differences in COVID-19 immune response are not fully understood (44). T-cell activation at the early phase of SARS-CoV-2 infection is robust in older female patients with COVID-19, whereas it declines with age and has worse COVID-19 outcomes in only male patients with COVID-19 (45). Male sex is a risk factor for COVID-19 severity and death, likely because it enhances viral fusion and multiplication (20). It would explain why men are more likely to have complications, require ICU admission and mechanical ventilation, and have higher mortality than women, regardless of age (46). Although women have a more robust ability to control infectious agents (45), the adaptive immune system of men appears more susceptible to the adverse effects of virus impact (47, 48). To the best of our knowledge, this is the first report of the sex-dependent differences of biomarkers of systemic inflammatory response, such as NLR, MLR, and PLR.

### Perspectives and Significance

Severe and non-surviving patients affected by COVID-19 had a higher NLR and MLR, and a lower MPR. The sensitivity of NLR, MLR, and PLR to predict severity was better in men (69–77%), whereas their specificity was enhanced in women compared to men (70–76 vs. 23–48%). High NLR, MLR, PLR, and low MPR levels were predictors of COVID-19 severity with different performance in men and women. Sex-dependent differences in immune responses related to COVID-19 disease would pave the way to explain why current worldwide statistics show more men than women dying of SARS-CoV-2 infection.

### Limitations

This study was a retrospective and single-center observational analysis with no patient follow-up. Patient stratification was based on PaO_2_ because chest tomographies were time-consuming. Although respiratory frequency data were not available, categorization accuracy was proven by pulse oximetry (49). Hemoglobin oxygen saturation in the non-severe group was almost normoxic (94.7 ± 2.1%), whereas it was below the recommended range for patients with COVID-19 in the severe group (88.1 ± 3.3%). Finally, the effects of comorbidities, hormonal supplementation, and gender-dependent lifestyles (e.g., smoking and drinking habits) were considered speculative (46, 50) and therefore beyond the scope of this study.

## Conclusion

The NLR, MLR, PLR, and MPR levels were significantly higher in severe and non-survivors subjects. Except for PLR, the rest of the ratios were higher in men. There were also differences in most of these indicators between severe women and men and between non-survivor men and women. The sensitivity of NLR, MLR, and PLR was higher in men, who were at a higher risk of dying by COVID-19, whereas in women these biomarkers had higher cut-offs and enhanced specificity. Our findings confirm the validity of these parameters in predicting COVID-19 severity and incite a hematological analysis stratified by sex.

## Data Availability Statement

The raw data supporting the conclusions of this article will be made available by the authors, without undue reservation.

## Ethics Statement

The studies involving human participants were reviewed and approved by Hospital IESS Quito Sur. Written informed consent for participation was not required for this study in accordance with the national legislation and the institutional requirements.

## Author Contributions

SB, MF, MP-S, KC, EF-M, and HR conceived and designed the study. MP-S, KC, and FM collected the data. MP-S, SB, KC, FM, and MF analyzed the data. All authors reviewed and approved the final version of the manuscript.

## Conflict of Interest

The authors declare that the research was conducted in the absence of any commercial or financial relationships that could be construed as a potential conflict of interest.

## Publisher’s Note

All claims expressed in this article are solely those of the authors and do not necessarily represent those of their affiliated organizations, or those of the publisher, the editors and the reviewers. Any product that may be evaluated in this article, or claim that may be made by its manufacturer, is not guaranteed or endorsed by the publisher.

## Footnotes

1
https://www.covid19treatmentguidelines.nih.gov/overview/clinical-spectrum/

## Abbreviations

MLR: monocyte-to-lymphocyte ratio
MPR: mean platelet volume-to-platelet count ratio
NLR: neutrophil-to-lymphocyte ratio
PLR: platelet-to-lymphocyte ratio
AUC–ROC: area under the curve–receiver operating characteristic
RT-PCT: real-time reverse transcription-polymerase chain reaction
MPV: mean platelet volume
TLR: toll-like receptor
PSI: pneumonia severity index
IQR: interquartile range.
